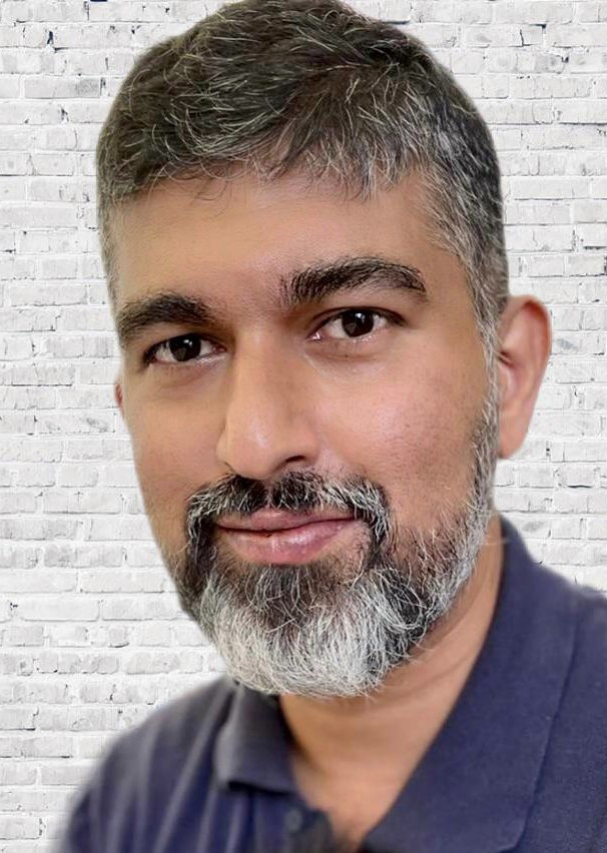# Effect of Surface‐Adsorbed and Intercalated (Oxy)anions on the Oxygen Evolution Reaction

**DOI:** 10.1002/anie.202207279

**Published:** 2022-08-04

**Authors:** J. Niklas Hausmann, Prashanth W. Menezes

**Affiliations:** ^1^ Department of Chemistry: Metalorganics and Inorganic Materials Technische Universität Berlin Straße des 17 Juni 135, Sekr. C2 10623 Berlin Germany; ^2^ Material Chemistry Group for Thin Film Catalysis—CatLab Helmholtz-Zentrum Berlin für Materialien und Energie Albert-Einstein-Str. 15 12489 Berlin Germany

**Keywords:** Electrode Materials, Oxygen Evolution Reaction, Oxoanions/Oxyanions, Water Splitting

## Abstract

As the kinetically demanding oxygen evolution reaction (OER) is crucial for the decarbonization of our society, a wide range of (pre)catalysts with various non‐active‐site elements (e.g., Mo, S, Se, N, P, C, Si…) have been investigated. Thermodynamics dictate that these elements oxidize during industrial operation. The formed oxyanions are water soluble and thus predominantly leach in a reconstruction process. Nevertheless, recently, it was unveiled that these thermodynamically stable (oxy)anions can adsorb on the surface or intercalate in the interlayer space of the active catalyst. There, they tune the electronic properties of the active sites and can interact with the reaction intermediates, changing the OER kinetics and potentially breaking the persisting OER *OH/*OOH scaling relations. Thus, the addition of (oxy)anions to the electrolyte opens a new design dimension for OER catalysis and the herein discussed observations deepen the understanding of the role of anions in the OER.

## Graphical abstract

(Oxy)anions can surface‐adsorb on and intercalate into oxygen evolution reaction (OER) electrocatalysts. There, they can affect the catalyst's electronic properties and interlayer spacing, stabilizing OER intermediates and potentially breaking the *OH/*OOH scaling relations, as described in this Minireview. Thus, adding (oxy)anions to the electrolyte opens a new OER design dimension.

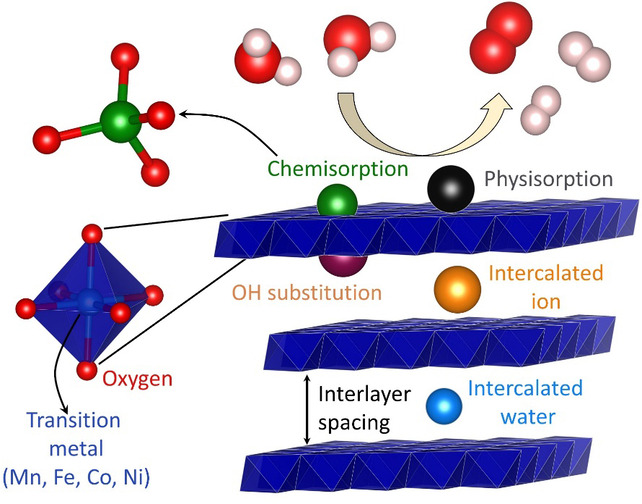

## 1 Introduction

For the complete decarbonization of our society, large‐scale electrocatalytic hydrogen production is required using regenerative electricity sources (solar, wind, hydro) and water.^[1]^ The obtained green hydrogen will first replace the fossil fuel based gray hydrogen in processes such as the ammonia and methanol synthesis and will later be used in new applications such as decarbonized steel production, marine transportation, aviation, and potentially large‐scale energy storage.^[1]^ The efficiency of electrocatalytic green hydrogen production is critically affected by the kinetically demanding oxygen evolution reaction (OER), which supplies the electrons and protons for the hydrogen evolution reaction (HER) and also for electrocatalytic CO_2_ or nitrogen reduction.[^2^, ^3^]

To find an ideal OER catalyst, a wide range of transition‐metal‐based materials have been investigated including several non‐oxide compounds.[^4^, ^5^, ^6^] However, under the harsh OER conditions, only certain oxidic phases are stable, e.g., layered nickel, iron, cobalt oxides/oxyhydroxides in alkaline media (we note that these phases, especially monometallic iron ones, potentially suffer from slow dissolution).[^4^, ^5^, ^6^, ^7^, ^8^, ^9^] For example, transition metal chalcogenides or pnictides will transform to transition metal oxides/oxyhydroxides, while the chalcogenide or pnictide anion (X^*n*−^) will be fully oxidized to the respective oxyanions (XO_4_^2−^ and XO_4_^3−^, see Pourbaix diagrams in reference [10]).[^4^, ^5^, ^6^] Due to the high water‐solubility of such oxyanions, they leach into the electrolyte.^[10]^ Thus, their role is that of a sacrificial reagent to form a disordered, porous, high‐surface‐area skeleton‐catalyst similar to the role of aluminum in the widely applied Raney nickel process.[^11^, ^12^, ^13^]

In 2020, an additional function of the anions besides being sacrificial reagent was reported, which is the surface‐adsorption of the thermodynamically stable, fully oxidized oxyanions.[^14^, ^15^] In subsequent reports, it was shown that these adsorbed species can stabilize the OER reaction intermediates (see Section 3 for a detailed discussion),[^14^, ^15^, ^16^, ^17^, ^18^, ^19^] and that they can enhance the activity of the most promising OER catalysts, such as nickel‐iron oxyhydroxides.^[16]^ Furthermore, this phenomenon adds a new, unexplored dimension into the OER catalyst parameter space that could finally break the persisting *OH/*OOH scaling relations.^[20]^ Before 2020, the intercalation of (oxy)anions into the interlayer space had been investigated already.^[21]^ This phenomenon is closely related to the surface‐adsorption, as it involves the same oxyanions in a similar proximity to the OER active sites.

This Minireview aims to critically discuss and interconnect the literature on the effect of intercalation and surface‐adsorption of (oxy)anions on the OER, which each or together have never been reviewed. We do not focus on synthetic aspects and strictly concentrate on the concepts that are in line with the thermodynamic stability of the materials (oxides/oxyhydroxides) and ions (oxyanions) under alkaline OER conditions (mainly Ni‐, Fe‐, Co‐based catalysts).^[22]^ We first provide an overview on the relevant host structures and the different potential interactions with the (oxy)anions. Afterwards, we comprehensively summarize in form of tables and critically discuss the effect of surface‐adsorbed or intercalated (oxy)anions on the OER. Finally, we provide clear conclusions on both phenomena, connect them, and give an outlook on future directions.

## 2 Structural Aspects of the Surface‐Adsorption and Intercalation of (Oxy)anions on/in Transition Metal Oxyhydroxides

### 2.1 The Structure of Transition Metal Oxyhydroxides

**Figure 1:**
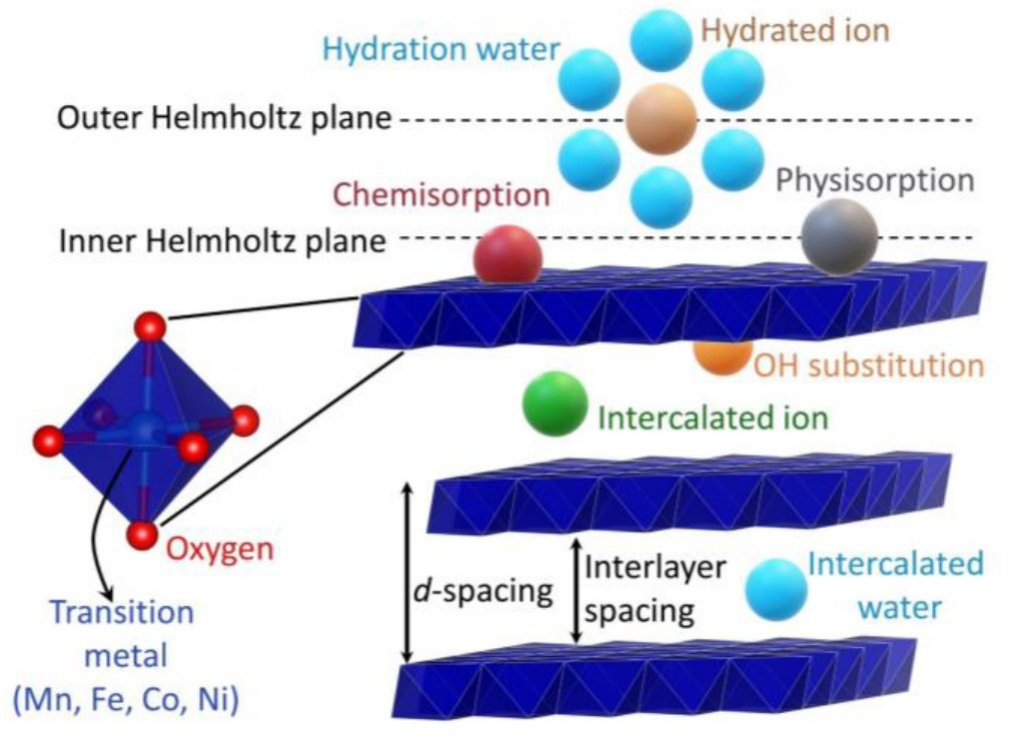
Overview of the different modes of interaction between (oxy)anions (colored spheres) and a layered, oxidic structure (blue layers, e.g., Fe/Co/Ni‐layered double hydroxides). The interaction modes are described in detail in Section 2.2.

Under alkaline oxygen evolution reaction conditions, the most prominent, earth‐abundant catalytic centers (Mn, Fe, Co, Ni) form layered, oxidic structures.[^3^, ^4^, ^5^, ^6^, ^10^] Figure 1 shows a model of such a structure where the blue layers comprise edge‐sharing [MO_6_] octahedra (shown on the left). These layers can be densely packed like in β‐MOOH in which the anionic layers are connected merely by protons, resulting in a small interlayer spacing (<5 Å).^[3]^ However, water and ions can intercalate into the layers, leading to larger interlayer spacings and modifying the catalytic properties.^[21]^ Furthermore, the layers can be small (molecular domains with 10–40 metal atoms) and do not necessarily stack parallel, leading to X‐ray amorphous phases.^[3]^

### 2.2 Different Modes of Adsorption and Intercalation

In Figure 1, the colored spheres represent different modes of interaction between the layered structure and ionic species or water in the double‐layer microenvironment.^[23]^ Those are:

1. Chemisorption, meaning that a chemical bond between the catalyst surface and the adsorbed species was formed. Such species are specifically adsorbed as their adsorption depends on the specific chemical properties of the adsorbate, e.g., a sulfate that is connected through a covalent bond to the surface metal center M−O−SO_3_.^[16]^
2. Physisorption, meaning that the electronic structure of the adsorbed species is not strongly perturbated and no new chemical bond is formed, e.g., water connected through hydrogen bonds or dioxygen through van der Waals interactions.[^23^, ^24^]
3. Electrostatic adsorption of hydrated (solvated) ions, meaning that the hydration shell is still intact, which limits the minimum distance between the catalyst surface and the ion. e.g., [K(H_2_O)_*n*_]^+^.
4. Intercalated ions that are in the interlayer space influencing the spacing, e.g., carbonate bond through hydrogen bonds with the layers’ OH groups.^[25]^
5. Substitution of the hydroxide groups by other species inside the layers and at the surface by covalent bonding, e.g., methoxy, replacing M−OH with M−OMe bonds.[^26^, ^27^]

## 3 The Effect of Surface‐Adsorbed Oxyanions

### 3.1 Reconstruction and Thermodynamic Stability of Oxyanions

While the most prominent catalytic centers (Fe, Co, Ni) form the described layered structures, the other components of the precursor (e.g., B, C, Si, N, P, S, Se, Mo…) are oxidized, as their oxidation potentials are dramatically lower than that of water (OER, 1.23 V vs reversible hydrogen electrode (*V*_RHE_)), e.g., at pH 13.89 (like in 1 M KOH)^[28]^ with a selenium concentration of 0.1 M [Eqs. (1)–(3)].^[10]^

(1) $$\mathrm{HSe}{}^{-}+\mathrm{OH}{}^{-}\to \mathrm{Se}+2\;e{}^{-}+H{}_{2}O\;0.20\;V{}_{\mathrm{RHE}},$$

(2) $$\mathrm{Se}+6\;\mathrm{OH}{}^{-}\to \mathrm{SeO}{}_{3}{}^{2-}+4\;e{}^{-}+3\;H{}_{2}O\;0.46\;V{}_{\mathrm{RHE}},$$

(3) $$\mathrm{SeO}{}_{3}{}^{2-}+2\;\mathrm{OH}{}^{-}\to \mathrm{SeO}{}_{4}{}^{2-}+2\;H{}_{2}O+2\;e{}^{-}\;0.88\;V{}_{\mathrm{RHE}}.$$

This leads to the formation of highly oxidized, electrolyte‐soluble oxyanions (e.g., B^III^O_4_^3−^, C^IV^O_3_^2−^, N^V^O_3_^−^, P^V^O_4_^3−^, S^VI^O_4_^2−^, MoO_4_^2−^…) that leach from the precursor. This leaching is important for the catalyst's properties and can lead to disordered, porous, high‐surface‐area skeleton‐catalysts that contain many accessible active sites and can be electrolyte‐penetrable and bulk active.^[3]^ This reconstruction process has been intensively reviewed elsewhere already.[^4^, ^5^, ^6^] In this Minireview, we focus on the reports that consider this reconstruction for the active phase catalyst (layered oxides) and the anions (oxyanions) and we do not discuss phenomena that were raised mainly on the assumption of no reconstruction, like anion regulation.^[29]^ It is worth mentioning here that, in a few reports, reduced chalcogenide species were found to be present after OER under academic testing conditions.[^30^, ^31^, ^32^, ^33^, ^34^] Even though these reports are interesting, they are not considered herein, as they do not provide sufficient proof that such species can survive under industrial conditions over months as required for application.

### 3.2 Ways to Introduce Surface‐Adsorbed Oxyanions

Looking at the reported reconstructed phases, even though the thermodynamically stable oxyanions are water/electrolyte soluble, in several reports, X‐ray photoelectron and Raman spectroscopy have identified traces of surface‐adsorbed oxyanions during and after OER on the catalyst.[^3^, ^15^, ^17^, ^35^, ^36^, ^37^, ^38^] It has been shown that the surface‐adsorbed oxyanions can originate from the following different sources:

1. They can arise from the oxidation of a precatalyst element with consecutive leaching and re‐adsorption, e.g., NiSe_2_ forms NiOOH and adsorbed SeO_4_^2−^_(aq)_ or Fe‐doped NiMoOOH forms Fe‐doped NiOOH and adsorbed MoO_4_^2−^_(aq)._[^15^, ^39^]
2. They can be added to the electrolyte, e.g., as SeO_4_^2−^_(aq)_ or a precursor such as SeO_3_^2−^_(aq)_.^[15]^
3. They can be formed during the synthesis prior to the OER reaction, e.g., the hydrothermal formation of sulfate adsorbed Co_3_O_4_.^[14]^

The origin of the oxyanions should not affect the way they surface‐adsorb, but a significant difference between the three options is that in the second case the concentration of the oxyanion in the electrolyte can be higher and precisely controlled.

The surface adsorption of oxyanions is a reversible chemisorption process, and the oxyanions will only remain if they possess a similar or lower adsorption energy than other competing species such as OER intermediates. Density functional theory (DFT) calculations revealed that the adsorption energy is often negative (e.g., −1.9 eV for SeO_4_^2−^ on NiOOH)^[15]^ and occurs at the same transition metal sites where the OER intermediates are adsorbed (Ni preferred to Fe).[^16^, ^39^] Thus, when too many oxyanions are present, they will block the OER active sites.[^23^, ^40^, ^41^] This competition is consistent with the observation that the addition of oxyanions to the electrolyte first leads to an improvement of the OER activity (see following paragraphs), but after a certain concentration (in the order of magnitude of 0.1 M) the overpotential increases.[^15^, ^16^] In this regard, it is worth mentioning that anodic potentials can increase the local concentration of (oxy)anions in the (near‐)surface area of the electrode, as oxyanion migration into the double‐layer can take place to compensate the build‐up positive charge on the electrode surface.^[42]^

### 3.3 The Effect of Surface‐Adsorbed Oxyanions on the OER

**Figure 2:**
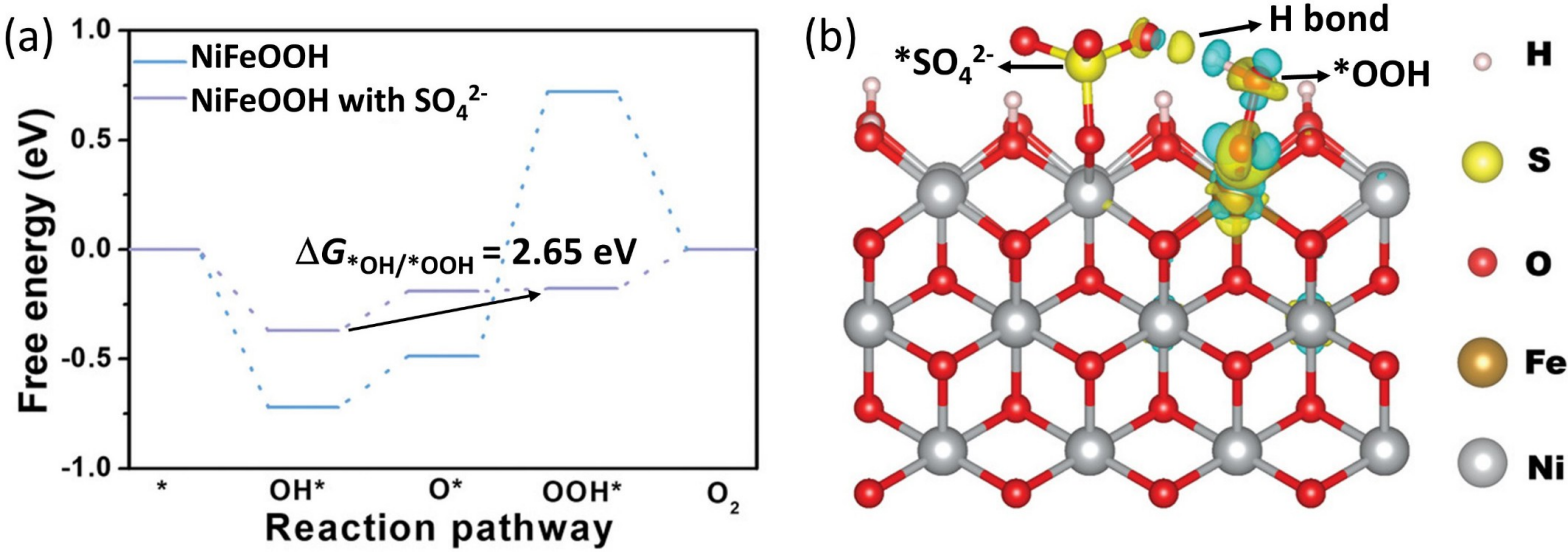
a) Gibbs free energy diagram of the OER intermediates at an applied potential of 1.23 V over NiFeOOH and NiFeOOH with surface‐adsorbed sulfate. In Rossmeisl and co‐workers’ initial report on the OER scaling relations, the energy difference between the *OH and *OOH intermediates was found to be always around 3.2 eV, which is significantly higher than the ideal 2.46 eV and would correspond to a minimum overpotential of 370 mV.^[20]^ The energy difference in (a) is only 2.65 eV which would correspond to a minimum overpotential of merely 95 mV, for an ideal O* adsorption strength.^[43]^ b) The interaction between the *OO−H intermediate and surface‐adsorbed −O−[SO_3_] forming a hydrogen bond. The hydrogen bond that can be formed with the *O−H intermediate (not shown here) is weaker and longer, and thus leads to a relative stabilization of the *OOH intermediate compared to the *OH one.^[14]^ Both images are taken and modified from ref. [16].

As a first overview, Table 1 lists the catalysts and surface‐adsorbed species that have been reported for the OER so far. Until recently, surface‐adsorbed oxy)anions have received little attention. However, in 2020, Zhang and co‐workers reported that sulfate can be surface‐adsorbed during OER and enhances the OER activity of Co‐ and mixed CoFeNi‐based electrocatalysts.^[14]^ Their report includes DFT simulations assuming a common OER reaction pathway (H_2_O→*OH→*O→*OOH→O_2_), indicating that the sulfate stabilizes the *OOH intermediate with respect to the *OH one (see Figure 2 for the stabilizing interaction on NiFeOOH).^[16]^ The possibility of this stabilization was later confirmed by other reports.[^16^, ^17^, ^19^, ^39^] This is of particular importance, as a universal scaling relation between these two intermediates had been identified with an energy difference larger than ideal.^[20]^ In this regard, for many catalytic systems, a stabilization of the *OOH intermediate leads to improved OER kinetics,^[43]^ and is highly desired but extremely challenging, as changing the electronic properties of a flat catalyst will lead to a similar stabilization or destabilization of the *OH and *OOH intermediates.^[20]^ The surface‐adsorbed oxyanions can act as a second binding site, forming hydrogen bonds of different strengths with the *OH and *OOH intermediates due to geometrical differences (Figure 2).[^14^, ^16^, ^39^] The first spectroscopic evidence that a surface‐adsorbed oxyanion interacts with the *OOH intermediate was provided for RuFeO_*x*_ with adsorbed sulfate.^[17]^ In this report, *in‐situ* infrared spectroscopy finds that the *O−OH stretching vibration is redshifted by 25 cm^−1^ in the presence of sulfate, indicating a weakened *O−OH bond and proving an interaction. In short, the surface‐adsorption of oxyanions with suitable geometry and electronic properties provides a design possibility to stabilize OER intermediates and to break the *OH/*OOH scaling relations.

**Table 1:** Overview on the reports that discuss the effect of adsorbed oxyanions on the OER in detail. Sorted chronologically by the year of publication.

(Pre)catalyst phase^[a]^	Adsorbed oxyanion	Comments	Ref.
Co_3_O_4_	SO_4_^2−^	DFT shows that *OH/*OOH scaling relations can be broken through adsorption	[14]
CoNiFeO_x_	SO_4_^2−^		[14]
NiSe_2_	SeO_4_^2−^	First example of *in‐situ* Raman investigation and addition of oxyanion to the electrolyte	[15]
Ni(OH)_2_	(S/Se)O_4_^2−^		[15]
Cu(OH)_2_	SeO_4_^2−^		[15]
Co(OH)_2_	SeO_4_^2−^		[15]
NiS_2_	SO_4_^2−^		[15]
NiFeOOH	PO_4_^3−^	First non‐chalcogenide oxyanion	[46]
CoFeMoO_*x*_/MoS_*x*_/SO_4_^2−^	SO_4_^2−^		
RuFeO_x_	SO_4_^2−^	Infrared spectroscopy identifies interaction with *OOH intermediate	[17]
NiFeOOH	SO_4_^2−^/CrO_4_^2−^/HCO_3_^−^	First report on non‐chalcogenate adsorbed oxyanions	[16]
NiFe‐LDH	SO_4_^2−^	Chloride oxidation suppression through sulfate addition	[49]
ZnIn_2_S_4_	SO_4_^2−^	Photocatalytic OER	[53]
MoNiFeOOH	MoO_4_^2−^	Time‐resolved tracking of re‐adsorption after leaching	[39]
NiFeS	SO_4_^2−^		[54]
Co(Zn)OOH	SO_4_^2−^		[55]
FeNiOOH	PO_4_^3−^	Effect of adsorption on d‐band center is analyzed by DFT	[47]
Ni_3_S_2_	SO_4_^2−^	“	[48]
NiOOH	(P/S/Se)O_x_	First application for methanol oxidation, the oxyanions facilitate methanol and hydroxide adsorption	[18]

[a] For the precise stoichiometry and structure of the catalytic phases as well as reconstruction details, the referenced reports must be consulted.

Besides the direct interaction with the OER intermediates, other potential influences of the surface‐adsorbed oxyanions relevant for the OER have been identified:

1. Oxyanions are bases and can act as proton acceptors or carriers, which is especially important in (near‐)neutral or only mildly alkaline electrolytes.[^38^, ^44^, ^45^]
2. The chemisorption of oxyanions changes the electronic structure of the oxyanion and the catalyst. In this regard, DFT calculations have shown that surface‐adsorbed oxyanions can increase the density of states at the Fermi level,[^16^, ^39^, ^46^] and that the d‐band center can be changed, potentially resulting in optimized OER intermediate adsorption energies.[^18^, ^47^, ^48^]
3. Surface‐adsorption of sulfate has been shown to create a negatively charged surface layer, which repulses other anions such as chlorine and thus can prevent unwanted side reactions such as chlorine oxidation in direct seawater splitting.[^49^, ^50^]
4. In metal–organic frameworks, oxyanions from the electrolyte have also been shown to adsorb in the vicinity of the active site influencing catalysis.[^51^, ^52^]

## 4 The Effect of Intercalated (Oxy)Anions

### 4.1 The Dynamic Nature of (Oxy)Anion Intercalation

Layered, oxidic host‐structures of the promising, earth‐abundant OER catalytic centers (Mn, Fe, Co, Ni) can be synthesized with a wide range of intercalated species.[^22^, ^56^] Intercalation into the interlayer space is often a reversible process, which makes it crucial for applications like batteries or supercapacitors.[^57^, ^58^] Thus, under prolonged testing, an intercalated species will be exchanged, if an alternative thermodynamically more favorable replacement is available. For example, during alkaline OER, hydroxide and carbonate (from CO_2_ from air) are present in the electrolyte. Such an exchange can result in the collapse of the as‐synthesized structure and might be understood as a part of the (often beneficial) OER reconstruction process.[^4^, ^5^, ^6^, ^59^, ^60^] Furthermore, it is critical to consider the redox properties of the intercalated anions, as the harsh OER conditions usually lead to the transformation of most anions to their fully oxidized oxyanions, when they are in contact with the electrolyte.^[10]^ Therefore, any report on intercalation must consider possible catalyst/anion reconstruction and apply *in‐situ*/post‐OER characterizations to deduce meaningful conclusions. Furthermore, when an anion is added to the electrolyte it can either surface‐adsorb or intercalate. Thus, the two reversible processes cannot be viewed independently and are competing.

Regarding the exchange of intercalated species during the OER, in general, it was observed that the intercalation of divalent anions is usually thermodynamically more favorable than intercalation of monovalent anions.[^61^, ^62^] For the OER, in 2016, a pioneering work by Müller and co‐workers was published including the formation of NiFe layered double hydroxides (LDH) with 10 different intercalated anions (NO_3_^−^, BF_4_^−^, F^−^, Cl^−^, I^−^, ClO_4_^−^, C_2_O_4_^2−^, PO_4_^3−^, SO_4_^2−^, CO_3_^2−^).^[61]^ After exposure to 1 M KOH, in all cases, the anion was exchanged for carbonate stemming from dissolved, ambient CO_2_. The exchange of anions with ambient carbonate has also been observed by others,[^62^, ^63^, ^64^, ^65^, ^66^] and found to occur as well for larger molecules such as dicarboxylates,^[59]^ organosulfates,[^64^, ^65^] and polyoxometalates.^[66]^ Due to this exchange, Müller and co‐workers found the same OER performance for the ten different NiFe‐LDH starting compounds under non‐inert conditions.^[61]^ Therefore, when testing an intercalated catalyst, one must always consider the exchange of carbonate and possibly hydroxide.

**Figure 3:**
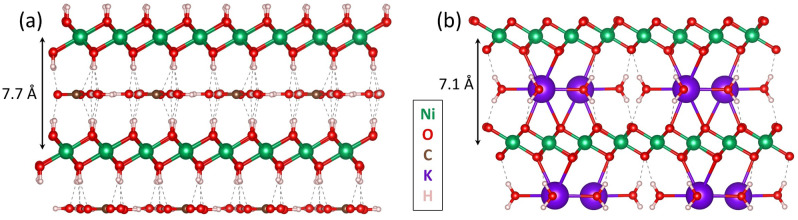
The layered structures determined by Dionigi and co‐workers for Ni, NiFe, and CoFe‐LDH.^[25]^ Hydrogen bonds are shown as dashed lines. a) The alpha structure with intercalated water and carbonate (M_8_CO_3_(OH)_16_⋅4 H_2_O) that is present at potentials below the OER onset. b) The deprotonated gamma structure with intercalated water and potassium M_8_K_2_O_16_⋅4 H_2_O that forms under OER conditions and is the OER catalytically active phase.

While carbonate intercalation is easy to track by X‐ray photoelectron or infrared spectroscopy, the intercalation of hydroxide can hardly be identified, as hydroxide has similar spectroscopic/physical/chemical properties as water and the hydroxide groups of the host structure. Nevertheless, as hydroxide is ubiquitous in alkaline electrolyte, it has been speculated that it intercalates to a certain extent and might even replace carbonate.[^61^, ^63^, ^67^] But what does such an intercalation look like and does it really occur? In this regard, an important aspect is that hydroxide is a strong base and potentially can deprotonate the hydroxide groups of the host LDH structure. In such a scenario, hydroxide intercalation is actually the intercalation of water coupled with the deprotonation of the host hydroxyl groups. By combining electrochemical measurements, operando X‐ray scattering, and absorption spectroscopy with DFT calculations, Dionigi and co‐workers recently answered this question conclusively for NiFe‐ and CoFe‐LDH.^[25]^ They found that, in ambient 1 M KOH, without applied OER potential, the LDHs adopt an alpha structure similar to hydrotalcite ((Co/Ni)_6_Fe_2_CO_3_(OH)_16_⋅4 H_2_O), which contains intercalated water and carbonate ions that are bonded to the host structure by hydrogen bonds (see Figure 3a). Under applied OER potential, the transition metals are further oxidized and the hydroxide groups of the host structure are completely deprotonated in the presence of KOH_(aq)_. The resulting absence of hydrogen bonds makes the carbonate intercalation unfavorable, and instead, a fully deprotonated gamma phase with potassium and water intercalation is formed (Co/Ni)_6_Fe_2_K_2_O_16_⋅4 H_2_O (see Figure 3b). Thus, (alkaline) hydroxide can replace carbonate, especially under OER conditions; however, it does not “intercalate” like other anions (weaker bases) and deprotonates the host structure with concomitant alkali ion and water intercalation.

### 4.2 The Effect of Intercalated (Oxy)Anions on the OER

Following the above discussion of the dynamic nature of intercalation and catalyst reconstruction, we present an overview on OER reports dealing with intercalation in Table 2. Almost all reported host materials are Fe/Co/Ni‐LDH. A wide range of anions have been intercalated into these hosts, including inorganic species like oxyanions, halides, fluorinated species, and comparably large polyoxometalates and carbon nanotubes as well as organic species such as alkoxides, carbonic acids, organosulfates, amides, and aldehydes. As discussed above, most of these species are oxidized or exchanged during OER. Nevertheless, some reports have shown that the detour of *in‐situ* OER interlayer species exchange by carbonate or alkaline hydroxide can be beneficial for the catalytic activity, and as such *in‐situ* formed catalysts have been demonstrated to be more active than the same directly synthesized carbonate intercalated materials.[^59^, ^60^, ^63^, ^64^, ^68^, ^69^, ^70^, ^71^] This is especially the case for large intercalated species that strongly increase the *d*‐spacing like dodecylsulfate (*d*‐spacing>20 Å),[^64^, ^65^, ^69^] PW_12_O_40_^3−^ (*d*‐spacing>11 Å),^[66]^ benzoate (*d*‐spacing>14 Å),[^68^, ^72^, ^73^] or large dicarboxylic acids (e.g., C_10_ sebacic acid *d*‐spacing>19 Å).^[59]^ Compounds with such large interlayer spacing usually have higher surface areas than their carbonate counterparts, leading to more available catalytically active sites. Furthermore, the large *d*‐spacing can enable/enhance electrolyte‐penetrability/mass‐transport in the interlayer space, enabling phenomena like bulk OER activity.[^3^, ^60^, ^65^, ^69^, ^74^] Such compounds with large *d*‐spacing can then show activity similar to that of their exfoliated counterparts.^[64]^ Obviously, the *in‐situ* OER exchange of the large anions by e.g. carbonate will reduce the interlayer spacing and thus shrink the volume/surface area; however, traces of the large molecules often remain trapped and the resulting catalysts usually still have larger surface areas, a higher porosity, and more catalytically accessible sites than the directly synthesized carbonate‐intercalated counterparts, as it has also been widely reported for other reconstruction processes.[^4^, ^5^, ^6^, ^59^, ^60^] We note here that for changes of smaller interlayer spacings (7.4–8.6 Å) no correlation with the OER activity could be deduced.^[61]^

**Table 2:** Overview on the reported anion intercalated oxidic materials for the OER (sorted alphabetically).

Intercalated species	(Pre)catalyst phase^[a]^ and reference	Comments
Acetaldehyde C_2_H_4_O	CoFe‐LDH^[81]^	
Alkoxides C_*n*_H_*m*_O^−^	NiFe LDH,^[82]^ CoFe‐LDH,^[81]^ NiEt_2_,^[26]^ Co(Fe)OHMe^[27]^	Alkoxides likely oxidize under OER conditions,^[83]^ alkoxide can replace the OH groups of LDH[^26^, ^27^]
Benzoate C_7_H_5_O_2_^−^	Co‐LDH,[^68^, ^73^] Ni‐LDH^[72]^	*d*‐spacing >14 Å
Biuret C_2_H_5_N_3_O_2_	CoFe‐LDH^[81]^	
Borate BO_4_^3−^	NiFe‐LDH[^60^, ^74^]	
Carbon nanotubes	NiFe‐LDH^[84]^	
Carbonate CO_3_^2−^	Co‐LDH,^[68]^ NiFe‐LDH,[^59^, ^60^, ^61^, ^63^, ^69^, ^70^, ^76^] CoFe‐LDH,^[81]^ CoNi LDH^[85]^	In basic solution, carbonate from ambient CO_2_ replaces most other intercalated species[^61^, ^64^, ^66^]
Chlorine oxyanions ClO_n_‐	NiFe‐LDH[^61^, ^77^]	
Citrate C_6_H_5_O_7_^3−^	Co‐LDH,^[86]^ NiFe‐LDH^[87]^	
Dicarboxylates C_*n*_H_*m*_O_4_^2−^	Co‐LDH,^[73]^ NiFe‐LDH[^60^, ^61^, ^77^]	Exchange by borate and carbonate during OER^[60]^
Formamide (CH_3_)_2_NCHO	NiFe‐LDH^[88]^	Formamide likely oxidizes under OER conditions^[89]^
Halides X^−^	CoFe‐LDH[^61^, ^64^, ^77^, ^81^, ^90^]	Easily replaced by carbonate[^61^, ^64^, ^66^]
Molybdate MoO_4_^2−^	NiFe‐LDH,[^91^, ^92^, ^93^] ZnFe‐LDH^[94]^	
Nitrate NO_3_^−^	Ni‐LDH,^[95]^ NiCeO_*x*_H_y_,^[95]^ Co‐LDH,^[86]^ NiFe‐LDH,[^61^, ^63^, ^66^, ^90^, ^93^] ZnFe‐LDH,^[94]^ CoNi‐LDH^[85]^	*In‐situ* Raman shows that nitrate leaves the structure during OER^[95]^
Nitrite NO_2_^−^	NiFe‐LDH^[77]^	Nitrite likely oxidizes under OER conditions^[10]^
Organosulfates C_*n*_H_*m*_SO_4_^2−^	Co‐LDH,^[86]^ NiFe‐LDH^[69]^	*d*‐spacing >32 Å,^[65]^ IR shows replacement by carbonate at prolonged OER^[65]^
Organosulfonates C_*n*_H_*m*_SO_3_^−^	NiCo‐LDH,^[96]^ NiFe‐LDH^[80]^	Includes large calixarenes^[96]^
Peroxydisulfate S_2_O_8_^2−^	NiFe‐LDH^[77]^	Peroxydisulfate likely oxidizes under OER conditions^[10]^
Phosphorus oxyanions H_*x*_PO_*y*_^*z*−^	NiFe‐LDH[^61^, ^76^, ^77^]	OER activity follows redox potential (PO_4_^3−^<HPO_3_^2−^<H_2_PO_2_^−^),^[76]^ HPO_3_^2−^ and H_2_PO_2_^−^ likely oxidizes under OER conditions,^[10]^ phosphate easily replaced by carbonate^[61]^
Polyoxometalates	NiFe‐LDH^[66]^	IR shows replacement by carbonate during OER^[66]^
Sulfate SO_4_^2−^	NiCo‐LDH,^[96]^ NiFe‐LDH[^61^, ^64^, ^65^, ^71^, ^90^]	Easily replaced by carbonate^[61]^
Sulfite SO_3_^2−^	NiFe‐LDH^[77]^	Sulfite likely oxidizes under OER conditions^[10]^
Tetrafluoroborate BF_4_^−^	NiFe‐LDH^[61]^	Easily replaced by carbonate^[61]^
Thiosulfate S_2_O_3_^2−^	NiFe‐LDH^[77]^	Thiosulfate likely oxidizes under OER conditions^[10]^
Tungstate WO_4_^2−^	NiFe‐LDH^[70]^	
Vanadate VO_4_^3−^	ZnFe‐LDH,^[94]^ NiFe‐LDH^[93]^	

[a] For the precise stoichiometry and structure of the catalytic phases as well as reconstruction details, the referenced reports must be consulted.

The large *d*‐spacing does not affect the intrinsic activity of the active sites, as those are likely the edge sites of the layers.^[75]^ However, the interlayer species can also directly affect the OER active sites by

1. changing the electronic properties of the active site through electron withdrawing or donating effects,
2. deprotonating an intermediate or acting as a proton transfer reagent or base,
3. stabilizing OER intermediates as it was shown for surface‐adsorbed species.

**Figure 4:**
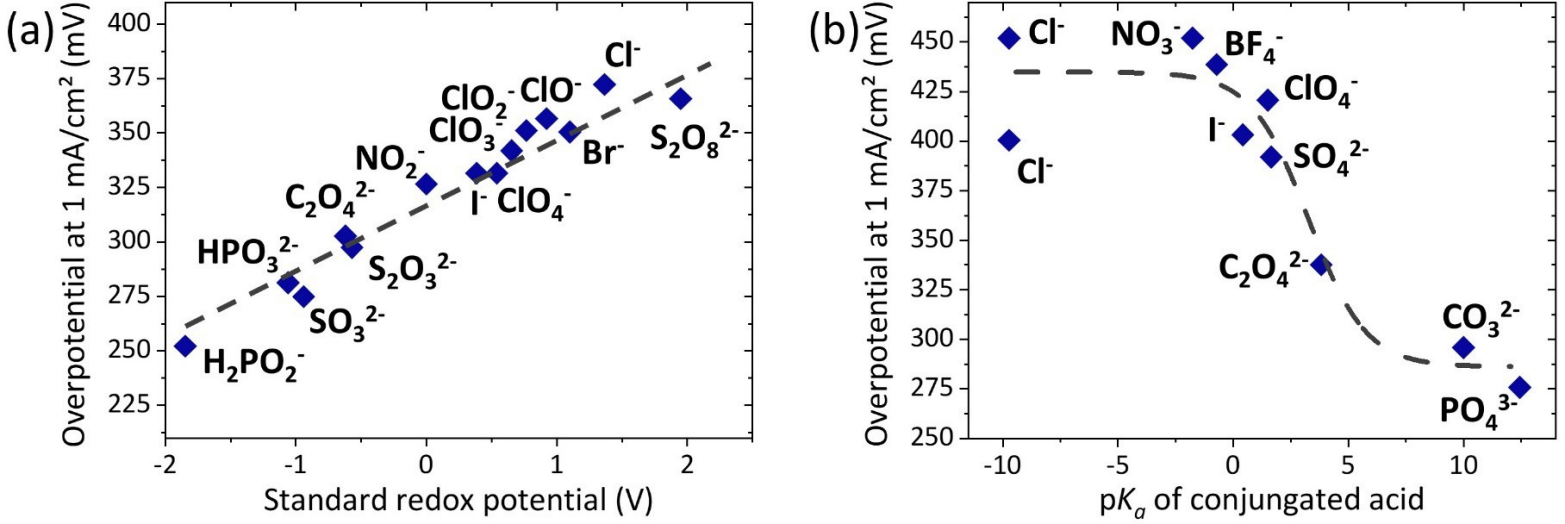
a) The linear relation between the redox potential (electron donation) of the interlayer species and the OER activity as observed by Sun and co‐workers.[^76^, ^77^] b) The sigmoidal correlation between the basicity of the interlayer species (p*K*_a_ of conjugated acid) and the OER activity as observed by Müller and co‐workers.^[61]^

Point 1 has been investigated by Sun and co‐workers.[^76^, ^77^] They prepared NiFe‐LDH with 16 different intercalated anions and found that the redox potential correlates with the OER activity, whereas anions that are stronger reducing agents lead to higher activities. This observation is explained by the electron‐donating properties of the anions. The stronger reducing agents (the less oxidized anions, e.g., ClO^−^ vs ClO_4_^−^) donate more electrons to the Ni and Fe sites of the LDH. This donation could also be observed in the X‐ray photoelectron spectra of Fe and Ni through a negative shift of the binding energies for stronger reducing properties of the anion and by DFT‐derived Baader charges of Ni and Fe. Sun and co‐workers claim that the higher electron density at the LDH metal centers makes it easier to oxidize them and thus enhances the OER activity. The correlations they find between redox potential/binding energy/Baader charge and the OER activity are remarkable (Figure 4a) and this phenomenon undoubtedly deserves more attention, even though the opposite trend based on a smaller set of anions has also been reported.^[78]^ Open questions that remain are that the anions leading to high OER activities should all be oxidized under OER conditions and mostly be exchanged. This is not what Sun and co‐workers observe after around 9 h of continuous operation, which they explain with the circumstance that the anions are in the bulk of the structure and thus not in contact with the electrolyte and cannot be oxidized/exchanged. Nevertheless, it is questionable if this observation will also be made after prolonged industrial OER conditions.

Point 2 is crucial for (near‐)neutral water splitting,[^45^, ^79^] where a strong base is missing. However, this Minireview focuses on the alkaline OER, where hydroxide is present in the electrolyte. Nevertheless, in 1 M KOH_(aq)_, a sigmoidal correlation between the basicity of the interlayer species and the OER activity has been found by Müller and co‐workers (Figure 4b).^[61]^ They hypothesize that this correlation is possible because the hydroxide content within the layers might be low and thus other, weaker bases have to perform the deprotonation of water and OER intermediates that are required for the formation of dioxygen. The observation that bases weaker than hydroxide can be involved in the OER was also confirmed by Sun and co‐workers.^[80]^ For an nonafluoro‐1‐butanesulfonate‐intercalated NiFe‐LDH, they revealed by a combination of pH‐dependent kinetics investigations, chemical probing, proton inventory studies, and isotopic and atom‐proton‐transfer measurements that the intercalated sulfonate serves as proton transfer mediator deprotonating water in the rate‐determining step and thus improving the OER kinetics.^[80]^

Point 3 has not been described in the intercalation literature so far but has been widely observed for surface‐adsorbed oxyanions (see Section 3.3). As it has been shown that intercalated species can serve as proton mediators during the OER, they are in the vicinity of the intermediates and thus can potentially stabilize them.

## 5 Conclusion and Outlook

(Oxy)anions can surface‐adsorb on or intercalate into the most active alkaline OER catalysts and improve their catalytic properties. Both surface‐adsorption and intercalation involve the same (oxy)anions and both phenomena are highly dynamic during the OER. Thus, regardless of the as‐prepared phase, the species that is surface‐adsorbed or intercalated might be a completely different one and is a function of the electrolyte (pH, addition of (oxy)anions), the applied potential, and the reconstructed phase. When an anion is in the electrolyte, it can either surface‐adsorb or intercalate, and thus the two highly reversible phenomena cannot be viewed independently and are competing. In both cases, the (oxy)anions are in the direct vicinity of the active sites. Therefore, they can interact with the OER reaction intermediates by stabilizing them or by transferring protons. In this way, they enhance the OER kinetics and can potentially break the *OH/*OOH scaling relations. Furthermore, surface‐adsorbed and intercalated anions are usually connected to the active sites through a bridging oxygen atom or through strong hydrogen bonds. This chemical interaction can change and fine‐tune the electronic properties of the active sites/structure. The interaction with the OER intermediates and the active sites of (oxy)anions deepens the understanding of the role of anions of precatalysts for the OER and could be the key to explain previous observations while opening a new and relatively unexplored design strategy.

The potential of the herein discussed approaches is significant and broad, as the (oxy)anions merely must be added to the electrolyte, and thus they can be used to manipulate and fine‐tune in principle every OER catalyst. However, the studies on these effects are still in their infancy and the dynamic behavior of the (oxy)anions during OER makes it challenging to obtain meaningful data by conventional methods, slowing down the research progress. In this regard, currently, it is not clear, which (oxy)anions are most suitable for which catalysts and why. Also, the adsorption/intercalation energies for most (oxy)anions on catalysts under OER conditions are unknown. Systematic studies involving and interconnecting *in‐situ* observations and DFT simulations are needed to deepen the understanding of the enhanced activity and enable (oxy)anion‐based catalyst design. So far, the surface‐adsorbed and intercalated (oxy)anions were characterized by X‐ray photoelectron, infrared, and Raman spectroscopy, while only the latter was applied *in‐situ*. For intercalated species, also *in‐situ* X‐ray diffraction methods could be utilized, as they determine the *d*‐spacing, which is strongly related to the nature of the intercalated (oxy)anions. For further development of the field, more *in‐situ* characterization methods must be exploited such as X‐ray absorption spectroscopy on the anion edges, which could finally provide geometrical information on the bonding situation of the (oxy)anion. Furthermore, besides anions, also cations can surface‐adsorb or intercalate into the interlayer space and thus might have similar effects on the OER.[^97^, ^98^] This is particularly important as every anion comes with a cation, and to date, it is unclear if the addition of, e.g., sodium sulfate has the same effect as potassium or magnesium sulfate. We anticipate that this critical Minireview will provide guidance for the analysis of these phenomena and will motivate and help researchers to participate in this novel and exciting OER research direction.

## Conflict of interest

The authors declare no conflict of interest.

## Biographical Information

*Jan* *Niklas Hausmann is currently pursuing his Ph.D. degree under the supervision of Dr. P*. *W. Menezes and Prof*. *Dr. M. Driess at the Department of Metalorganics and Inorganic Materials, Technical University of Berlin (Germany). Prior to this, he worked on superconductivity in the group of Yoshiteru Maeno at Kyoto University (Japan). His current research mainly focuses on the oxygen evolution reaction with the application of various in‐situ and ex‐situ methods to deduce structure–activity relations*.

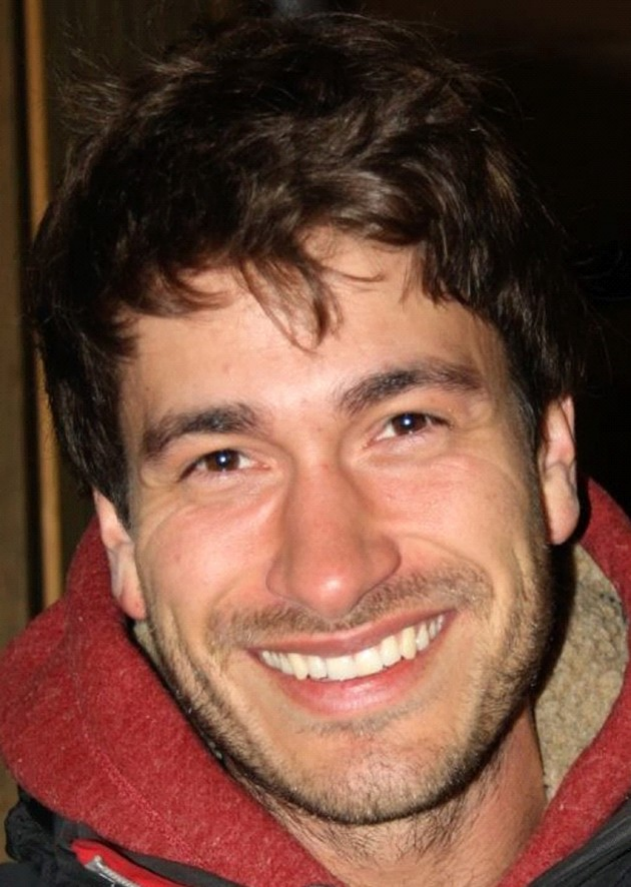

## Biographical Information

*Prashanth* *W. Menezes is a head of materials chemistry for thin‐film catalysis group at CatLab of the Helmholtz‐Zentrum Berlin für Materialien und Energie and inorganic materials group at Technische Universität Berlin. He received his Ph.D. from Max Planck Institute for Chemical Physics of Solids in Dresden, following which he moved to Technische Universität München and then to Technische Universität Berlin to work on energy catalysis. His research focuses on the design, development, and structural understanding of novel unconventional catalysts in heterogeneous catalysis, especially in the area of redox oxygen catalysis, (photo)electrocatalytic water splitting as well as electrochemical redox reactions*.